# HGK-sestrin 2 signaling-mediated autophagy contributes to antitumor efficacy of Tanshinone IIA in human osteosarcoma cells

**DOI:** 10.1038/s41419-018-1016-9

**Published:** 2018-09-26

**Authors:** Jia-Hau Yen, Sheng-Teng Huang, Hung-Sen Huang, Yi-Chin Fong, Yi-Ying Wu, Jen-Huai Chiang, Yuan-Chih Su

**Affiliations:** 1 Research Cancer Center for Traditional Chinese Medicine, Department of Medical Research, China Medical University Hospital, Taichung, Taiwan; 2 Department of Chinese Medicine, China Medical University Hospital, Taichung, Taiwan; 3 School of Chinese Medicine, China Medical University, Taichung, Taiwan; 4 Department of Orthopaedics and Traumatology, China Medical University Hospital, Taichung, Taiwan; 5 Department of Sports Medicine, College of Health Care, China Medical University, Taichung, Taiwan; 6 Department of Surgery, China Medical University Beigang Hospital, Yunlin, Taiwan; 7 Department of Medical Laboratory Science and Biotechnology, China Medical University, Taichung, Taiwan; 8 Management Office for Health Data, China Medical University Hospital, Taichung, Taiwan; 9 Graduate Institute of Integrated Medicine, School of Chinese Medicine, China Medical University, Taichung, Taiwan

## Abstract

Tanshinone IIA (TIIA) is a diterpenoid naphthoquinone isolated from the herb *Salvia miltiorrhiza* with antitumor effects manifested at multiple levels that are mechanistically obscure. In our previous studies, we illustrated that TIIA treatment triggered apoptosis in human osteosarcoma 143B cells both in vitro and in vivo, accompanied with mitochondrial dysfunction. Importantly, the overall survival rate of patients with osteosarcoma who were randomly recruited to *S. miltiorrhiza* treatment was significantly higher than those without. Pursuing this observation, we evaluated the potential effect of TIIA on autophagy induction in osteosarcoma both in vivo and in vitro. We discovered that TIIA inhibited osteosarcoma cell survival through class I PI3K and Akt signaling pathways. In contrast, expression of class III PI3K required in the early stages of autophagosome generation was predominantly enhanced by TIIA treatment. Our study indicated that treatment of TIIA effectively induced autophagy in human osteosarcoma cells, which contributed to the blockade of anchorage-independent growth of osteosarcoma cells and ameliorated tumor progression in NOD/SCID mice. We demonstrated that TIIA-mediated autophagy occurred in a sestrin 2 (SESN2)-dependent but not Beclin 1-dependent manner. In addition, we defined the activation of HGK (MAP4K4 or mitogen-activated protein kinase kinase kinase kinase)/SAPK/JNK1/Jun kinase pathways in upregulating transcription of SESN2, in which TIIA triggered HGK/JNK1-dependent Jun activation and led to increased Jun recruitment to AP-1-binding site in the SESN2 promoter region. Our results offer novel mechanistic insight into how TIIA inhibits osteosarcoma growth and suggest TIIA as a promising therapeutic agent for the treatment of cancer.

## Introduction

Osteosarcoma, a highly aggressive tumor arising in long bones, is the most commonly occurring primary malignancy in teenagers and young adults, with a broad spectrum of morphologies. Peak incidence of osteosarcoma occurs during the adolescent growth spurt, and the fact that it occurs primarily in the areas of active bone growth and repair, suggests genetic and molecular alterations that disrupt osteoblast differentiation are important in the etiology of the disease^1^. Current treatment strategy involving chemotherapy in combination with aggressive surgical resection has greatly improved the survival rates of patients with osteosarcoma. However, recurrence still occurs at the rate of 30–40%, while the 10-year survival rate is decreased by 20–30% with lung metastasis^2^. The development of effective, nontoxic therapeutic strategies using active natural substances with proven anticancer qualities may offer more promising preventive and therapeutic approaches for clinical application.

Danshen, the dried root of *Salvia miltiorrhiza* Bunge, is a well-known herb in traditional Chinese medicine (TCM) commonly used in clinical application as preventative and therapeutic remedies for coronary heart diseases, vascular diseases, stroke, hyperlipidemia, arthritis, hepatitis, and cancer^3–8^. Tanshinone IIA (TIIA), one of the most abundant constituents in the root of *Salvia miltiorrhiza*, exerts antioxidant and anti-inflammatory effects^9–13^. Although TIIA has been shown to induce G2/M growth arrest via downregulation of key cell-cycle regulatory protein CDC2 and cyclin B1 expression, it causes an apoptotic response in cancer cells^14^. In our previous study^15^, we found that TIIA administration resulted in a decrease in the mitochondrial fusion proteins, Mfn1/2 and Opa1, as well as an increase in the fission protein Drp1, which contributed to caspase cascade-mediated apoptosis in 143B osteosarcoma cells. These studies suggest that TIIA might be a promising agent for the prevention and/or treatment of osteosarcoma; however, a complete understanding of the underlying molecular mechanism of TIIA-mediated signaling networks in osteosarcoma growth inhibition remains wanting.

Autophagy, type II programmed cell death, which is initiated by numerous stresses, such as nutrient deprivation, hypoxia, intracellular reactive oxygen species (ROS) levels, viral and bacterial infection, oxidative stress, and chemical drugs, is an evolutionally conserved lysosomal process to recycle and degrade long-lived proteins and damaged cytoplasmic organelles in order to maintain cellar homeostasis and organismal health^16–18^. While moderate autophagy acts as self-protection against cytotoxicity^19^, in other cellular scenarios, consequent excessive autophagy may lead to cell death^20,21^. Current knowledge of the molecular intersections between the autophagic and apoptotic pathways is incomplete and fragmented. Thus, further investigations are needed into apoptosis-autophagy crosstalk, which may open the door to innovative and unique strategies for cancer therapy.

A markedly increased survival rate in patients with osteosarcoma who received Danshen has previously been confirmed. Thus, we designed the present study to further examine TIIA, the most abundant constituent of Danshen, and specifically the underlying molecular mechanisms behind TIIA-mediated inhibition of cancer cell growth. We elucidated that TIIA administration induced mitochondria dysfunction and autophagy in a SESN2 (sestrin 2)-dependent manner. Importantly, TIIA-induced autophagy was found to be essential for the TIIA inhibition of anchorage-independent cancer cell growth, demonstrating that autophagy induced by TIIA is cytotoxic to human osteosarcoma cells. The study furthermore uncovered a novel mechanism of TIIA in its activation of HGK/JNK1/c-Jun signaling cascades leading to SESN2 expression.

## Results

### TIIA treatment inhibited anchorage-independent growth of osteosarcoma cells and osteosarcoma progression in NOD/SCID mice

**Fig. 1:**
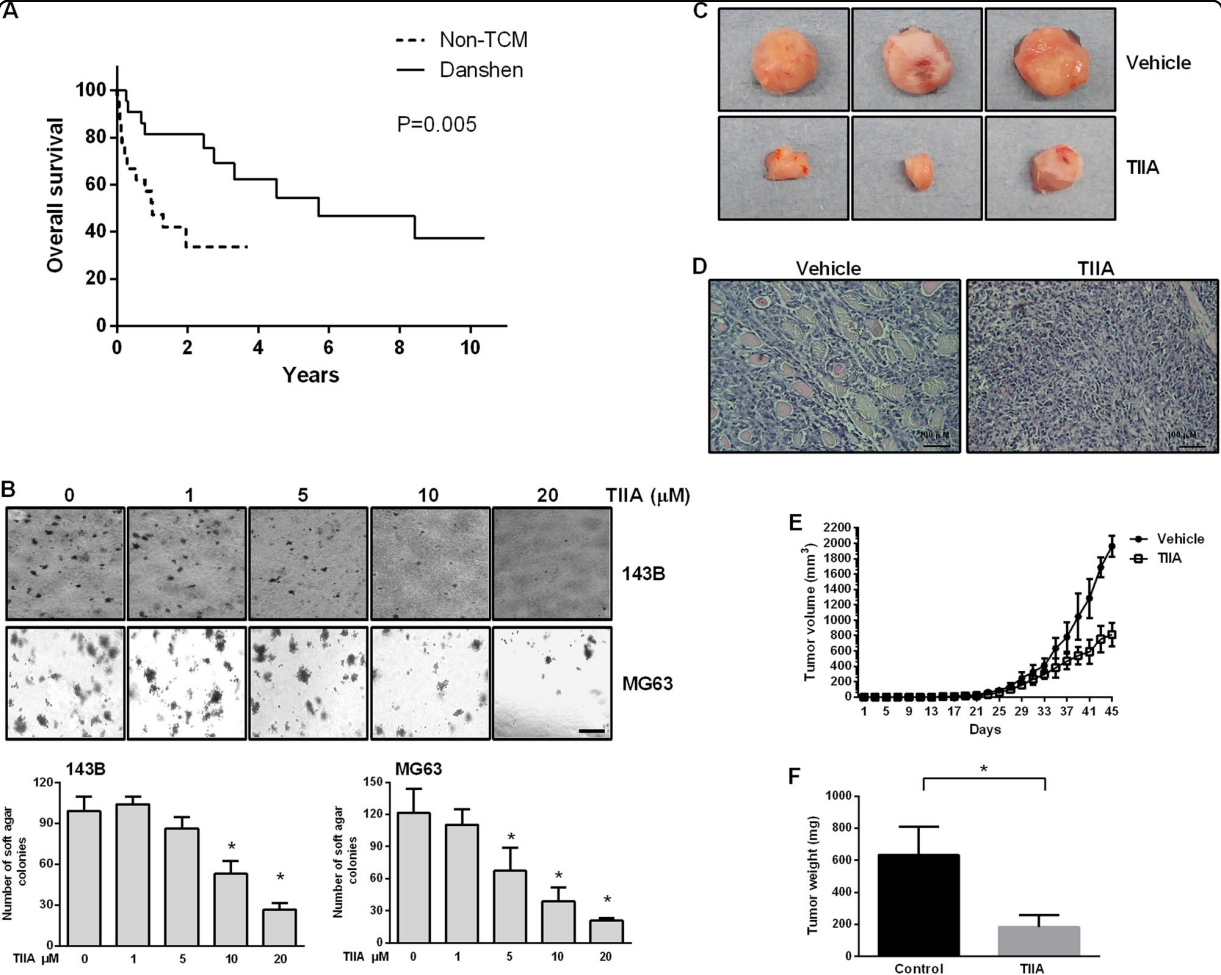
Danshen improved survival of patients with osteosarcoma and TIIA inhibited anchorage-independent growth in vitro and osteosarcoma growth in vivo. **a** Kaplan–Meier survival curves for osteosarcoma patients (*n* = 22; *P* = 0.005) with or without Danshen treatment. **b** Soft agar colony formation of osteosarcoma cell lines (143B and MG63) treated with various concentrations TIIA for 3 weeks. Histogram represented average number of colonies counted (in six microfields). Results were representative of three independent experiments. Scale bar: 500 μm. **c**–**f** 143B cell-derived tumors were developed in NOD/SCID mice and treated with TIIA or vehicle. Representative tumor images were photographed (**c**). Tumor growth was monitored by histological staining (H&E) (**d**) and measuring the tumor volume (**e**) and weight (**f**) for 45 days (*n* = 15 mice/group; *P < 0.05 compared with vehicle controls). The results were expressed as the means ± SD

Both the in vivo and in vitro anticancer activity of TIIA has been well-documented in our previous study^15^. Herein, we therefore further examined the long-term clinical outcome in patients with osteosarcoma who received Danshen treatment. Our study found the difference in mortality rates between the non-TCM patients and the Danshen-used group was highly significant (*p* = 0.005), indicating that Danshen treatment improved the survival rate of patients with osteosarcoma (Fig. 1a). As shown in Fig. 1b, the treatment of TIIA 10 μM resulted in an approximately 50% inhibition of anchorage-independent growth of both 143B and MG63 cells, while the higher concentrations were significantly more inhibited than other groups. For in vivo studies, 143B cells were implanted in the xenograft NOD.CB17-Prkdc^scid^/NcrCrl (NOD/SCID) mouse model, which has been comprehensively described in our previous study^15^. After administration of TIIA (20 mg/kg) every alternate day for 45 days, TIIA inhibited tumor growth (Fig. 1c), as well as abrogated the proliferation of osteoid in a high density of malignant cells under hematoxylin and eosin (H&E) staining (Fig. 1d). The tumor growth, including volume and weight, was significantly slower in TIIA-treated mice compared to those of the vehicle group (Fig. 1e, f). Collectively, these data demonstrated that TIIA exhibited potent antitumor activity both in vitro and in vivo.

### TIIA induced mitochondrial dysfunction and inactivation of the PI3K/Akt pathway in osteosarcoma cells

**Fig. 2:**
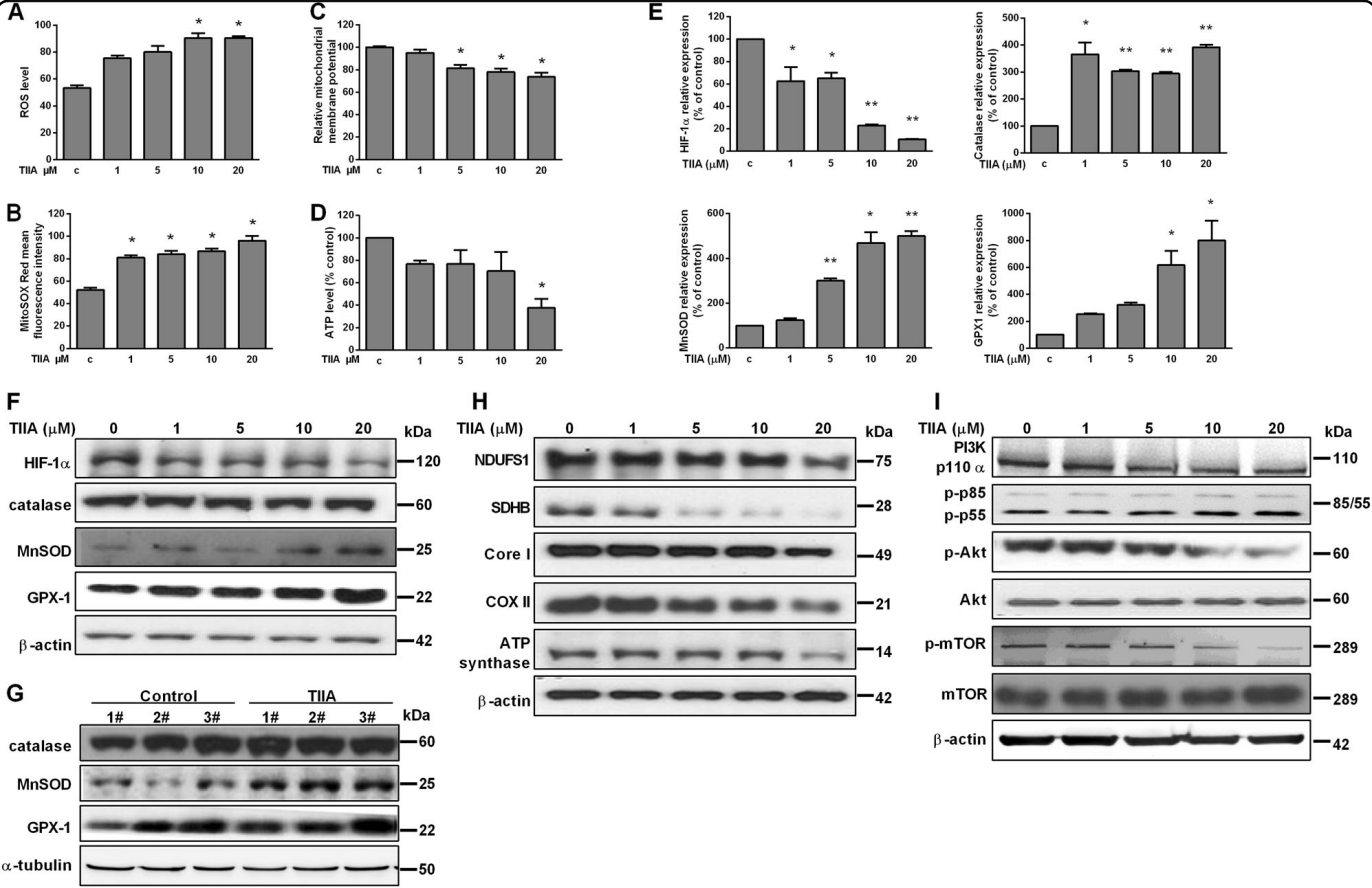
TIIA induced impairment of mitochondrial function and oxidative damage coupled with downregulation of PI3K/Akt/mTOR pathway. **a**–**d** 143B cells were treated with various concentrations of TIIA (1, 5, 10, and 20 μM) as indicated for 24 h and subjected to analysis of intracellular ROS (**a**), mitochondrial ROS (**b**), mitochondrial membrane potential (ΔΨm) (**c**), and ATP level (**d**). **e** HIF-1α, MnSOD, catalase, and GPX1 mRNA levels were measured by qRT-PCR in 143B cells treated with various concentrations of TIIA (1, 5, 10, and 20 μM) for 24 h. Data were normalized to the mean control group. The results were expressed as the means ± SD from three independent experiments (*n* ≥ 3, *P < 0.05, **P < 0.01 compared with untreated control). **f** Western-assisted analysis of HIF-1α, catalase, MnSOD, and GPX1 protein levels after TIIA treatment as indicated for 24 h in 143B cells. **g** 143B-derived tumors treated with TIIA or vehicle were subjected to immunoblotting analysis of catalase, MnSOD, and GPX1. **h** Western-assisted analysis of NDUFS1, SDHB, Core I, Cox II, and ATP synthase protein levels after TIIA treatment as indicated for 24 h in 143B cells. **i** 143B cells were treated with or without TIIA (1, 5, 10, and 20 μM) for 24 h, after which PI3K p110α, p85/p55, Akt, and mTOR phosphorylation were measured by western blot. β-actin and α-tubulin were served as loading control. Results were representative of three independent experiments

Based on our previous findings, which showed TIIA modulated mitochondrial morphological changes through fusion and fission dynamics^15^, we hypothesized the antitumor effect of TIIA was related to oxidative stress. To confirm and quantify the oxidative stress triggered by TIIA in 143B cells, identification of intracellular ROS level was analyzed by using oxidized 6-carboxy-2, 7-dichlorodihydrofluoroscein diacetate (DCFDA) with flow cytometry. As shown in Fig. 2a, we observed a significant increase in intracellular ROS levels at TIIA 10 μM following exposure for 24 h, but not continuously, increasing up to TIIA 20 μM. A parallel increase in mitochondrial ROS was observed by using the mitochondrial-specific probe MitoSOX Red (Fig. 2b). To further investigate the effect of TIIA on the physiological function of mitochondria, mitochondrial membrane potential (ΔΨm) and synthesis of ATP were evaluated. As expected, cells exposed to 24 h of TIIA 20 μM experienced a collapse of ΔΨm and ATP production (Fig. 2c, d). The primary endogenous antioxidant defense enzymes, such as MnSOD, detoxify reactive oxygen radicals to hydrogen peroxide, and then catalase and GPX1 enzymes catalyze its decomposition into water and oxygen^22^. In response to TIIA-induced ROS, we also found the transcription and translation of MnSOD and GPX1 were increased both in vitro and in vivo but catalase only increased in mRNA level (Fig. 2e–g). However, MnSOD has been reported to inhibit hypoxia-inducible factor-1α (HIF-1α) protein accumulation and DNA stability. In line with this, we found TIIA could downregulate HIF-1α mRNA and protein expression accompanied with enhanced MnSOD expression (Fig. 2e, f). The electron transport chain (ETC), or respiratory chain, is linked to proton movement and ATP synthesis. Thus, we examined the effect of TIIA on ETC, which consists of a series of large membrane-bound protein complexes (complexes I, II, III, and IV). As shown in Fig. 2h, we demonstrated a reduction in the expression of the ETC in complexes I, II, III, and IV, as well as ATP synthase (complex V).

Amongst the numerous pathways implicated in cancer development, the PI3K/Akt/mTOR signaling pathway is hyper-activated in cancer cells, which leads to cell survival and tumor proliferation^23–25^. Figure 2i demonstrated143B cells from the TIIA-treated group exhibited decreased expression of class I PI3K p110α, Akt, and mTOR but no significant change of P85 and P55, in comparison with the control group. Collectively, our findings suggested that TIIA-induced generation of ROS in mitochondrion was associated with mitochondrial dysfunction, and impairment of ETC reaction of the survival signaling pathway.

### TIIA effectively induced autophagy in human osteosarcoma cells

**Fig. 3:**
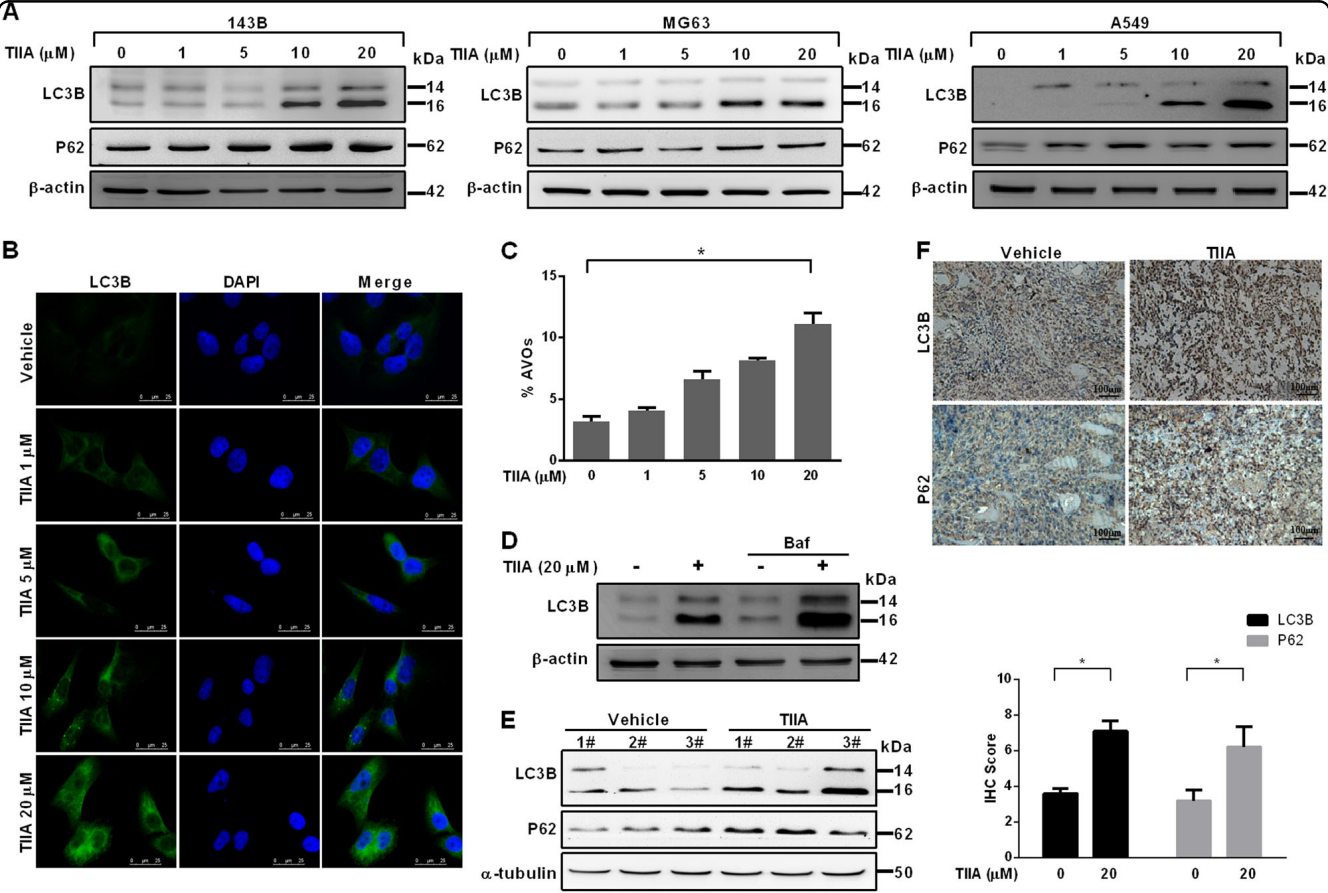
Induction of autophagy in human osteosarcoma cells following TIIA treatment. **a** Conversions of LC3B-I to LC3B-II were determined by immunoblotting following treatment with various concentrations of TIIA in human osteosarcoma 143B and MG63 cells for 24 h and human lung cancer A549 cells for 12 h, respectively. β-actin served as loading control. **b** Representative images for immunofluorescent staining of LC3B. 143B cells were treated with various concentrations of TIIA (1, 5, 10, and 20 μM) as indicated and subjected to immunofluorescence analysis of LC3B. Nuclei were stained with DAPI. **c** Quantification of cells developing AVO in 143B cells. AVO induction was examined by acridine orange staining and the percentage of developed AVOs was analyzed by flow cytometry. **d** 143B cells were pretreated with bafilomycin A1 (5 nM, 2 h) and then co-treated with TIIA (20 μM) for 24 h. The cells were extracted and the protein levels of LC3B were assessed by immunoblotting. **e** Western blot analysis of LC3B and P62 levels in 143B cell-derived tumors developed in NOD/SCID mice and treated with vehicle or TIIA for 45 days. **f** 143B-derived tumors treated with TIIA or vehicle were subjected to immunohistochemical analysis using LC3B and P62 antibodies. Bar diagrams, quantitation (IHC score) is based on percentage of cells showing expression of each protein from vehicle and TIIA-treated mice. β-actin and α-tubulin served as loading control. The results were expressed as the means ± SD from three independent experiments (*n* ≥ 3, *P < 0.05 compared with untreated control)

As we know that autophagy contributes to cell death when it exceeds normal homeostatic limits^26^, we then investigated whether TIIA induced autophagy in osteosarcoma cells. To detect the potential effect of TIIA on autophagy, we therefore treated human osteosarcoma cell lines, including 143B, MG63 cells, and human lung adenocarcinoma A549 cells, with different concentrations of TIIA for indicated times. As shown in Fig. 3a, TIIA treatment increased the amount of LC3B-II protein and upregulated P62 expression. Furthermore, we found that treating cells with TIIA resulted in a dose-dependent increase in LC3B puncta formation in 143B cells (Fig. 3b). Acidic vesicular organelle (AVO) formation (autophagosomes and autolysosomes) is also a characteristic feature of autophagy. Accordingly, the number of AVO-positive cells increased following TIIA treatment (Fig. 3c). To more specifically determine the role of TIIA in autophagy, we measured autophagy flux by treatment with bafilomycin A1, which inhibited lysosomal hydrolase activity and resulted in a subsequent block in autophagosome and lysosome fusion. TIIA treatment led to a further increase in LC3B-II levels in the presence of bafilomycin A1, indicating that TIIA increased autophagic flux (Fig. 3d and Supplementary Figure 1). Moreover, LC3 silencing reversed the reduction of apoptotic cells in TIIA-treated 143B cells (Supplementary Figure 2A–C). Both histological images and immunoblot of human osteosarcoma tissues were used to compare the accuracy of in vitro models. Tumors from the TIIA-treated group exhibited higher expressions of LC3B-II and P62 in comparison to the vehicle-treated group (Fig. 3e, f). Taken together, these results demonstrated that TIIA was capable of effectively inducing autophagy, which may contribute to the inhibition of anchorage-independent growth of human osteosarcoma cells.

### SESEN 2 played a critical role in TIIA-mediated autophagy and inhibition of anchorage-independent growth

**Fig. 4:**
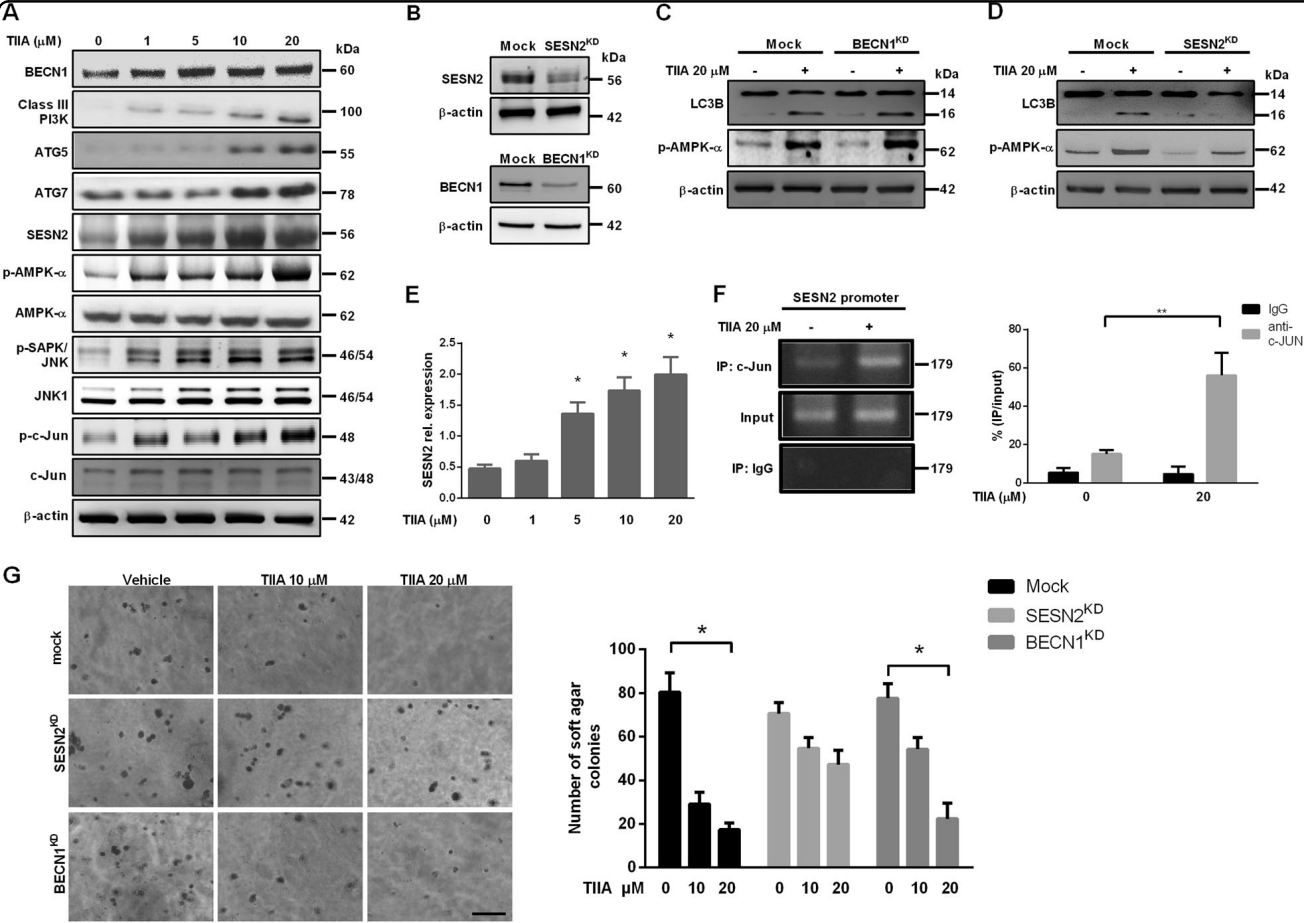
SESN2 but not Beclin 1 contributed to TIIA-induced autophagic induction and anchorage-independent growth inhibition in human osteosarcoma cells. **a** 143B cells were treated with various concentrations of TIIA (1, 5, 10, and 20 μM) as indicated for 24 h, after which BECN1, class III PI3K, ATG5, ATG7, SESN2, AMPK-α, p-AMPK-α, JNK1, p-SAPK/JNK, c-Jun, and p-c-Jun were measured by western blot. **b**–**d** shRNA SESN2 or shRNA BECN1 was stably transfected into 143B cells and the stable transfectants were identified (**b**). Following treatment with TIIA (20 μM) in 143B SESN2^KD^ (shSESN2) (**c**) and 143B BECN1^KD^ (shBECN1) (**d**) for 24 h, total lysates were immunoblotted for LC3B and p-AMPK-α. β-actin served as loading control. **e** SESN2 mRNA was measured by qRT-PCR in 143B cells treated with various concentrations of TIIA (1, 5, 10, and 20 μM) for 12 h. **f** 143B cells were treated with or without TIIA 20 μM for 12 h and subjected to ChIP assay using c-Jun antibodies. The purified DNA was analyzed by semiquantitative PCR (left) and real-time PCR (right) using specific primers spanning the c-Jun-binding sites on SESN2 promoter. ChIP DNA level was normalized to the level of input DNA. **g** Representative images of colonies of 143B SESN2^KD^ (shSESN2), 143B BECN1^KD^ (shBECN1), and 143B-mock (nonsense) cells in a soft agar colony formation assay in the absence or presence of various concentrations of TIIA were captured using a microscope Scale bar: 500 μm (left). Results were expressed as average number of colonies counted (in six microfields) (right). The results were expressed as the means ± SD from three independent experiments (*n* ≥ 3, *P < 0.05, **P < 0.01 compared with untreated control)

BECN1, which interacts with p53 to decide cell fate, is the crucial protein in modulation of autophagy and acts as a tumor suppressor^27^. It is interesting to note that SESN2 has also been linked to p53 and shown to modulate mTOR through AMP-activated protein kinase (AMPK)^28^. In a recent study, SESN2 is attributable to their capacity to promote the p62-dependent autophagic degradation during starvation^29^. In order to characterize the molecular mechanism underlying TIIA-mediated autophagic induction, we investigated whether BECN1 and SESN2 were involved in TIIA-mediated autophagy. As shown in Fig. 4a, TIIA treatment induced both BECN1 and SESN2 protein expression in a dose-dependent manner. Depending on the proteins recruited by BECN1, class III PI3K complexes and ATG complex differentially regulate the process of autophagosome formation. Herein, we detected that class III PI3K, ATG5, and ATG7 were increased after TIIA treatment. Meanwhile, the p-AMPK, p-SAPK/JNK, and p-Jun levels were significantly enhanced with the treatment of TIIA (Fig. 4a). The AMPK inhibitor BML-275 and JNK inhibitor SP600125 could effectively abrogate TIIA-mediated LC3B-II upregulation and reversed the cell proliferation downregulated by TIIA (Supplementary Figure 2D, E). To further determine whether BECN1 and/or SESN2 are key players in TIIA-induced autophagy, we used BECN1 and SESN2 short-hairpin RNA (shRNA) to knock down expression of endogenous BECN1 or SESN2 in 143B cells (Fig. 4b), and then investigated the impact on TIIA-induced LC3B-II expression and AMPK activation. Interestingly, immunoblot analysis for conversion of LC3 from LC3-I to LC3-II showed that knockdown of BECN1 did not inhibit TIIA-induced LC3-II and phosphorylation of AMPK-α expressions (Fig. 4c). However, knockdown of SESN2 significantly attenuated the TIIA-mediated induction of LC3-II and phosphorylation of AMPK-α (Fig. 4d). Consistent with the immunoblot analysis, we observed upregulation of the mRNA expression of SESN2 (Fig. 4e) and binding of Jun protein to the AP-1 site located in the SESN2 promoter upon treatment with TIIA (Fig. 4f). TIIA treatment effectively inhibited soft agar colony formation of mock and BECN1 knockdown osteosarcoma cells, but not of SESN2 shRNA cells (Fig. 4g). These results indicated that TIIA induced SESN2 expression, acting as an essential mediator of the signaling machinery used by TIIA in inhibiting the growth of osteosarcoma cells.

### TIIA inhibited growth of osteosarcoma via induction of autophagy in vivo

**Fig. 5:**
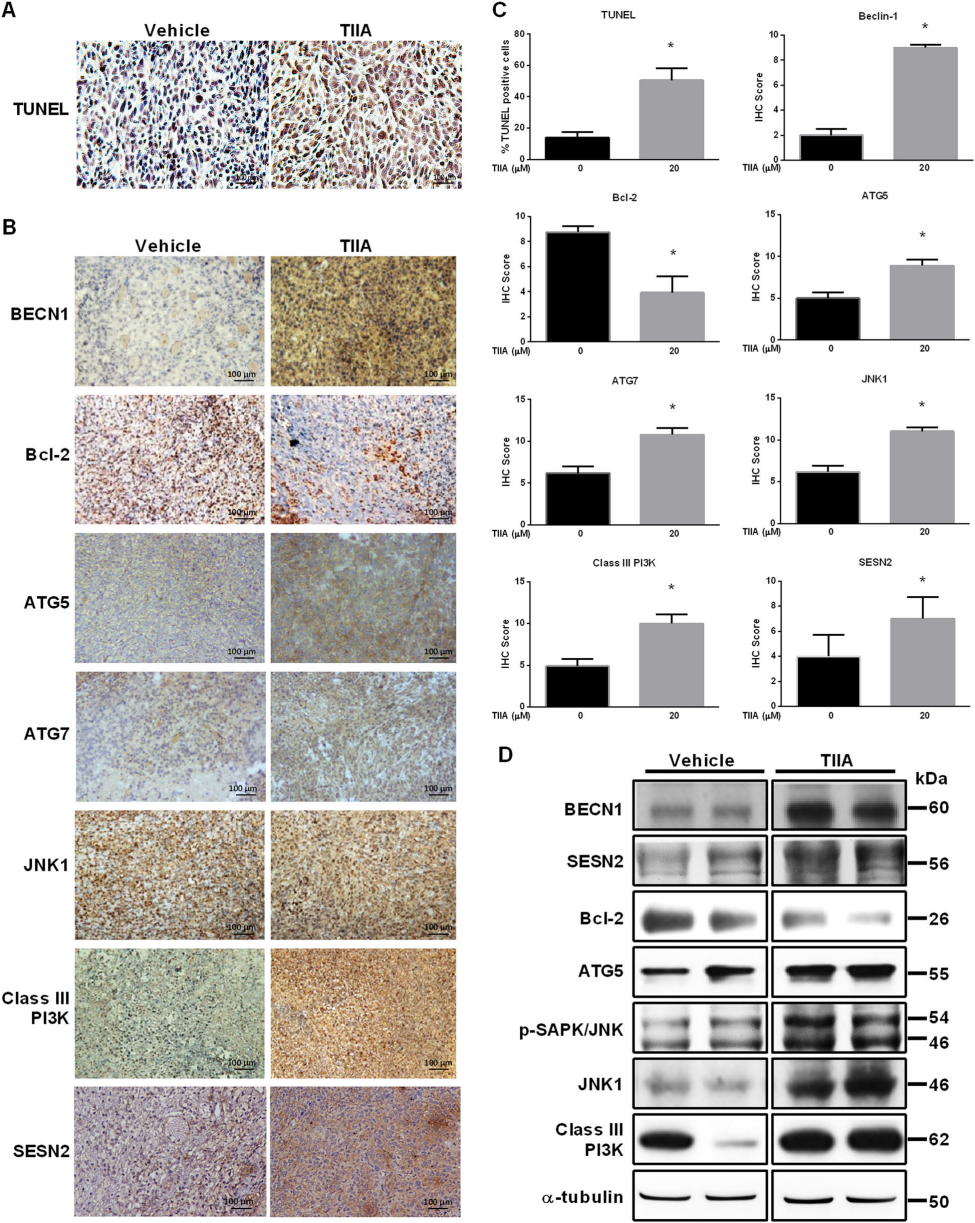
TIIA inhibited osteosarcoma xenograft growth in vivo. 143B cell-derived tumors were developed in NOD/SCID mice and treated with TIIA or vehicle for 45 days. **a** Detection of apoptosis in tumor tissues by TUNEL assay. **b** Expressions of BECN1, Bcl-2, ATG5, ATG7, JNK1, class III PI3K, and SESN2 were examined by immunohistochemistry. **c** The numbers of positive signals (dark blue) of TUNEL assay and IHC score were calculated and compared. **d** Western blot analysis of BECN1, SESN2, Bcl-2, ATG5, p-SAPK/JNK, JNK1, class III PI3K and SESN2 levels in 143B cell-derived tumors. α-tubulin served as loading control. The results were expressed as the means ± SD from three independent experiments (n ≥ 3, *P < 0.05 compared with untreated control)

As observed in vitro, the results confirmed that TIIA regulated osteosarcoma cell apoptosis via induction of autophagy. We therefore further investigated the in vivo physiological relevance of our in vitro findings by examining whether the autophagy pathway is integral for the inhibitory effects of TIIA on the development of osteosarcoma in xenograft osteosarcoma mouse models. As shown in Fig. 5a, TIIA-treated tumor tissues showed significant increase of TUNEL-positive cells. Meanwhile, immunohistochemistry (IHC) and western blotting showed the levels of autophagy-related Beclin 1, ATG5, ATG7, class III PI3K, SAPK/JNK phosphorylation, and SESN2 were upregulated after TIIA treatment, whereas the level of anti-apoptotic Bcl-2 was decreased (Fig. 5b–d). These results together verified that TIIA promoted tumor cell apoptosis in osteosarcoma by induction of autophagy both in vitro and in vivo.

### Hematopoietic progenitor kinase-germinal cell kinase-like kinase is the upstream kinase responsible for TIIA-mediated SESN2 induction

**Fig. 6:**
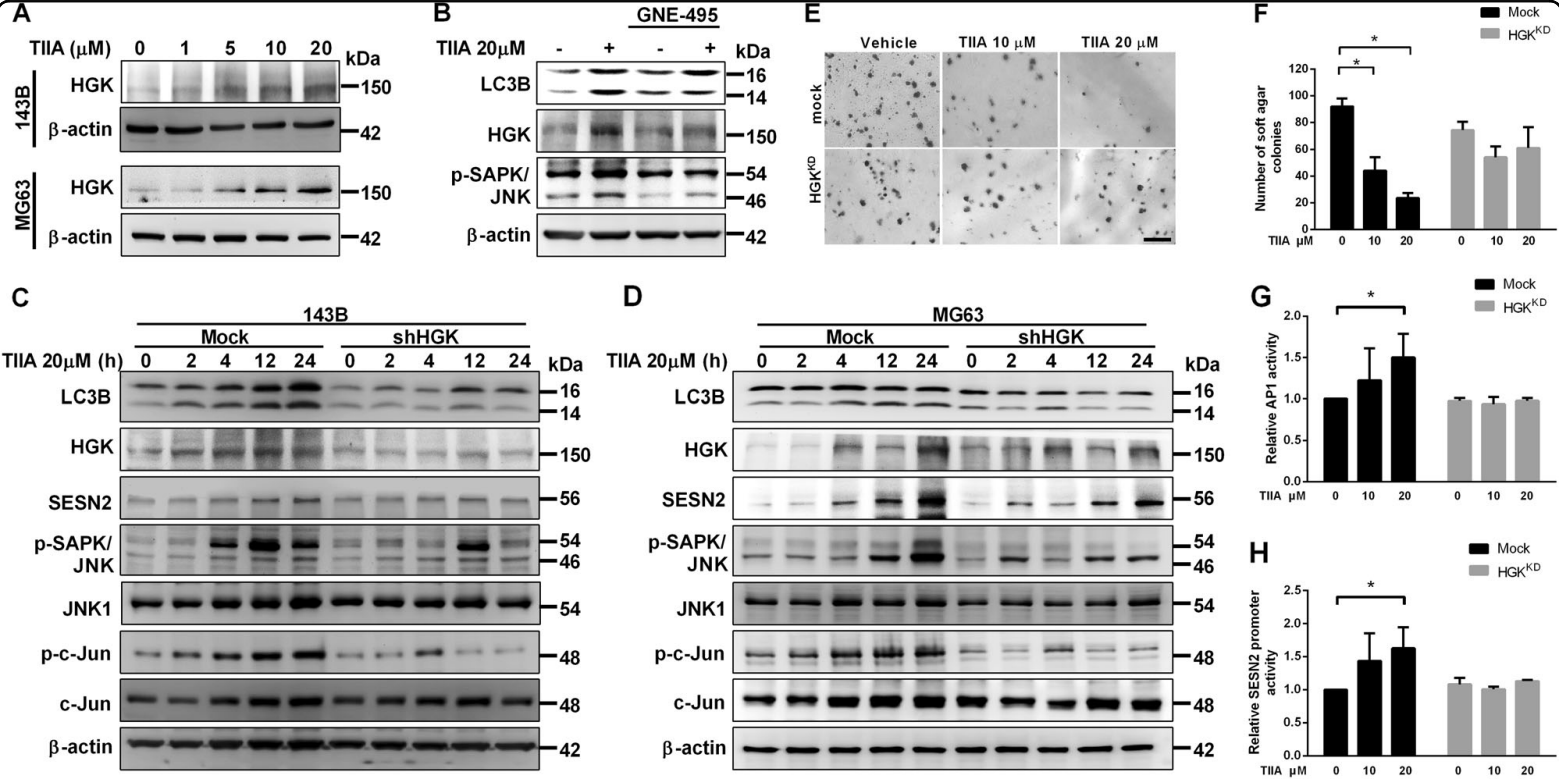
HGK acted an upstream regulator that mediated initiation of the JNK MAP kinase and SESN2-dependent autophagy following TIIA treatment. **a** Western-assisted analysis of HGK after TIIA treatment as indicated for 24 h in 143B and MG63 cells. **b** 143B cells were pretreated with GNE-495 (8 nM, 1 h) followed by TIIA treatment as indicated for 24 h. Total lysates were immunoblotted for LC3B, HGK, and p-SAPK/JNK expression. **c**, **d** shRNA HGK was stably transfected into 143B (**c**) and MG63 cells (**d**). Following treatment with TIIA (20 μM) for indicated time intervals, total lysates were immunoblotted for LC3B, HGK, SESN2, p-SAPK/JNK, JNK1, p-c-Jun, and total c-Jun expression. β-actin served as loading control. **e**, **f** Representative images of colonies of 143B-HGK^KD^ (shHGK) and 143B-mock (nonsense) cells in a soft agar colony formation assay in the absence or presence of various concentrations of TIIA were captured using a microscope. Scale bar: 500 μm (**e**). Results were expressed as average number of colonies counted (in six microfields) (**f**). **g**, **h** 143B cells were transiently transfected with the AP-1 luciferase reporter construct (**g**) or SESN2 promoter luciferase reporter construct (**h**). After 24 h, the cells were treated with various concentrations of TIIA for another 12 h and the relative luciferase activity was measured and presented as relative AP-1 activity or relative SESN2 promoter activity. The results were expressed as the means ± SD from three independent experiments (*n* ≥ 3, *P < 0.05 compared with untreated control)

Hematopoietic progenitor kinase-germinal cell kinase-like kinase (HGK), also known as MAP4K4, is a Ste20-related kinase, which leads to activation of JNK^30^. We therefore investigated whether HGK was the upstream kinase responsible for SESN2 induction by TIIA treatment. It is interesting to note that TIIA treatment resulted in a dose-dependent induction of HGK expression in osteosarcoma cells (Fig. 6a). To determine whether HGK was involved in TIIA-mediated autophagy, we used a selective HGK inhibitor, GNE-495, to block activity of HGK in vitro. Indeed, GNE-495 abrogated the expression of LC3B-II induced by TIIA, as well as p-SAPK/JNK and HGK (Fig. 6b). In a reciprocal approach, we employed shRNAs targeting human HGK to knock down expression of endogenous HGK in 143B and MG63 cells. As shown in Fig. 6c, d, we found HGK protein expression was significantly knocked down in HGKshRNA 143B and MG63 cells as compared with vector control cells. While HGKshRNA was applied to knock down endogenous HGK expression in both of the osteosarcoma cells, it not only blocked HGK activation but also attenuated p-SAPK/JNK/p-c-Jun and SESN2 induction and LC3-II formation following TIIA treatment. Moreover, TIIA treatment effectively inhibited soft agar colony formation of vector control cells, but not of HGKshRNA cells (Fig. 6e, f). These results indicated that HGK is crucial for TIIA-induced p-SAPK/JNK/p-c-Jun axis activation and SESN2-dependent autophagy. Intriguingly, we observed knockdown of HGK also inhibited the induction of endogenous AP-1-dependent transcriptional activity by TIIA (Fig. 6g), and further abolished the TIIA-mediated transcriptional induction of SESN2 promoter activity (Fig. 6h). Taken together, these results showed that TIIA-induced HGK overexpression was indeed a crucial component of the signaling machinery used by TIIA in inhibiting the growth of osteosarcoma cells.

**Fig. 7:**
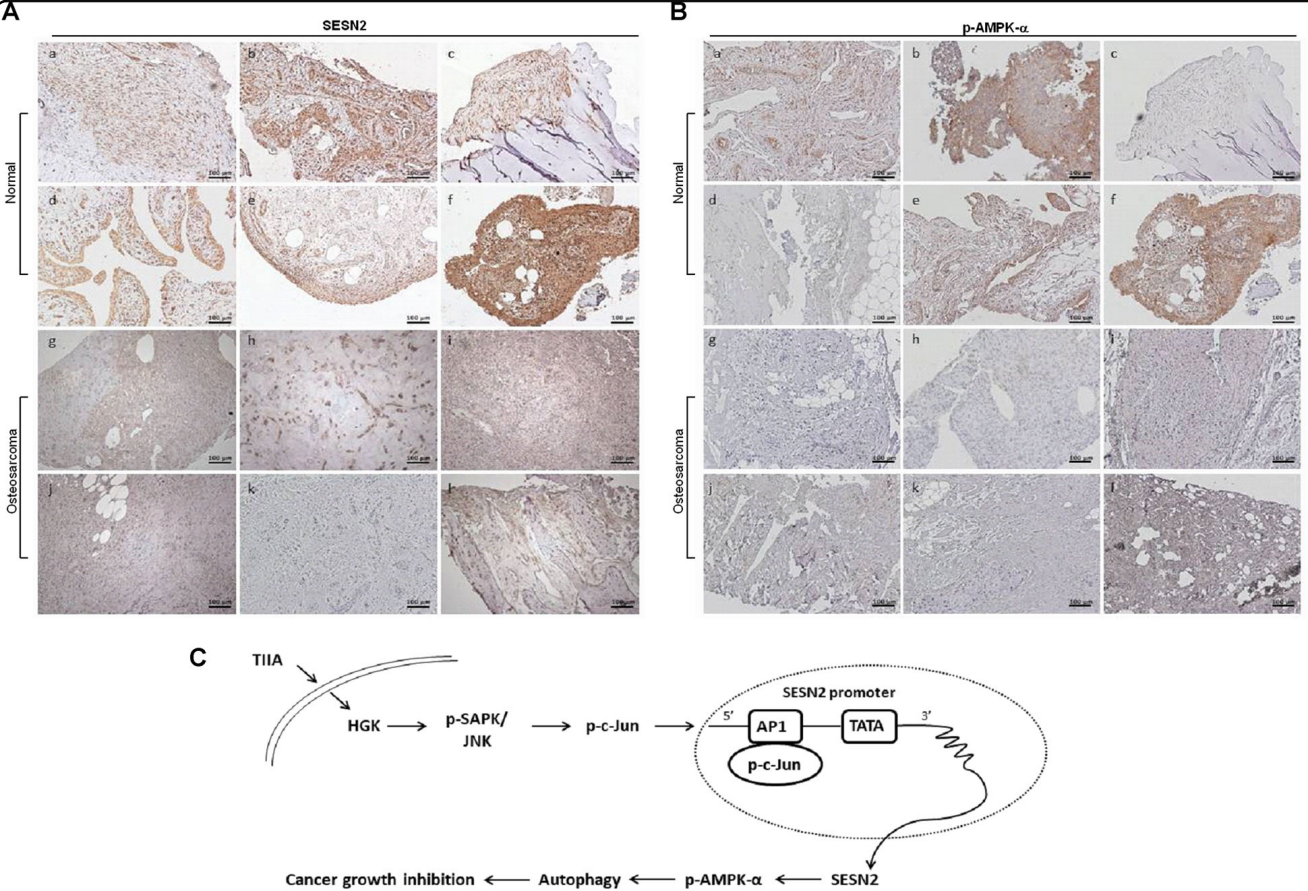
SESN2 and p-AMPK-α downregulation is a common event in osteosarcoma tissues. **a**, **b** Representative immunohistochemical staining of the expression of SESN2 (**a**) and p-AMPK (**b**) in normal bone tissues and osteosarcoma tissues. **c** Schematic model of TIIA-mediated induction of autophagic cell death in osteosarcoma cells

While the crucial role for the HGK-SESN2 axis in TIIA-mediated transcriptional induction of SESN2 in vitro has been determined, the level of SESN2 and its significance in human osteosarcoma remain unclear. To investigate whether SESN2 and p-AMPK proteins were expressed in human osteosarcoma, we examined bone sections of patients with osteosarcoma and normal bone tissue by immunohistochemistry. Strong staining of SESN2 was detected in normal bone tissue and was most strongly expressed in the sublining areas (Fig. 7a). p-AMPK was found mainly in the cytoplasm of normal bone tissue, while images C and D were negative or weakly stained (Fig. 7b). In contrast, SESN2 (Fig. 7a) and p-AMPK (Fig. 7b) staining were weak to absent in osteosarcoma tissue. Our results reveal that SESN2 and p-AMPK downregulation were associated with osteosarcoma formation, and further indicated that elevation of SESN2 and p-AMPK expression by TIIA could be related to its anticancer effects.

## Discussion

The awareness of the capacity of bioactive small-molecule agent TIIA to effectively inhibit carcinogenesis in a nontoxic and non-endocrine manner rendered this agent of potential interest in the treatment of osteosarcoma and sparked interest in investigating the underlying molecular mechanisms^15^. We found that TIIA effectively inhibited the growth of osteosarcoma cells both in vitro and in vivo and a provocative observation from this study led us to novel discoveries that extended beyond the popular proliferative and oncogenic roles of certain kinases and inhibition of these kinases by specific agents for tumor growth inhibition. HGK is known to be correlated with worse prognosis of several cancers^31–33^ and interact with Pyk2, which contributes to glioma cell migration^34^. Nevertheless, this study illustrates that TIIA effectively inhibits osteosarcoma growth by increased expression of HGK, which further upregulates the downstream target of SESN2. In the current study, the inhibition of HGK with both small-molecule inhibitor GNE-495 and shRNA-mediated gene silencing abolished TIIA-mediated SESN2 expression as well as autophagic induction. Our results elucidate an interesting mechanism by which TIIA-induced HGK/SESN2 results in concomitant upregulation/activation of LC3B-II and autophagy. In concurrence with these findings, we provide the schematic diagram of reciprocal signal axis regulation, which is operative in TIIA-induced SESN2-dependent growth inhibition of osteosarcoma cells (Fig. 7c).

The present study offers the first evidence of the capability of TIIA to activate HGK. Previous studies revealed that HGK specifically activated JNK, which in turn activated the transcriptional activity of c-Jun^35^. HGK had been implicated in the growth and migration properties of tumor cells^36^ and its expression level has been shown to closely correlate with clinical progression and poor prognosis among various tumor types^31–33^. In contrast, recent reports have also showed that HGK participates in negative regulation of mTOR phosphorylation^37^ and inducing the apoptotic cascade^38^. Importantly, the novel finding herein is the identification of HGK as a key protein in inducing autophagic cell death following TIIA treatment. The controversy may be due to the level of HGK and appears to involve coordinated induction of different elements between mitochondria and death receptor signal pathways.

Autophagy plays several fundamental roles, including supplying blocks for creation of new macromolecules and regulating energy status^39^. However, strong autophagy often leads to cell death, as it corresponds to a failed attempt to adapt to stress and survive. SESN2 is identified as a protein inducible upon DNA damage and oxidative stress^29,40^, and can suppress mammalian target of rapamycin complex 1 through activation of AMPK while accelerating autophagy^41^. It has been reported that SESN2 is able to cooperate with P62 to promote the autophagic degradation^29^. We looked for a link between SESN2 and the autophagy pathway and found that SESN2 promoted AMPK-α signaling and thereby upregulated autophagy accompanied with a light increase of P62 in osteosarcoma cells following TIIA treatment. The Warburg effect has been proposed as an adaptation mechanism characterized by high rates of glucose uptake and lactate production regardless of oxygen concentration to support the biosynthetic requirements of uncontrolled proliferation of many tumor cells^42^. In this scenario, AMPK, as a metabolic tumor suppressor, functions to coordinate glycolytic and oxidative metabolism in proliferating cells by restricting HIF-1α function^41^. In our observations, we found that the augmentation of p-AMPK-α levels by TIIA leads to decreased HIF-1α protein levels, which might be implicated in the inhibitory effect in anchorage-independent growth associated with TIIA-mediated intervention in metabolism. This proposal, further confirmed by the data, disclosed that mitochondrial dysfunction occurs concomitantly with the ROS generation and is unavailable in the mitochondrial electron transport. In the SESN2 knockdown analysis of the association between SESN2 and AMPK-α expression, we found that the low expression of SESN2 resulted in p-AMPK-α downregulation and inactivation of autophagy. We supposed that TIIA treatment induces autophagy via upregulation of p-AMPK-α in a SESN2-dependent manner, which further contributes to TIIA-mediated inhibition of anchorage-independent growth of osteosarcoma cells. By utilizing human osteosarcoma clinical specimens, we show that both SESN2 and p-AMPK (Thr172) are downregulated in a majority of patients with osteosarcoma, and low p-AMPK staining was reported to be correlated with poor prognosis of several cancers^43,44^. Herein, our study reveals that TIIA treatment induces SESN2/AMPK-α upregulation in osteosarcoma cells. Interestingly, Danshen, which is abundant with TIIA, also increased the overall survival rate in patients with osteosarcoma, consistent with observations of mouse xenografts models. These findings may suggest a positive impact of TIIA on the SESN2/AMPK-α signal axis for autophagic induction and repression of osteosarcoma development.

In conclusion, our data indicate the antitumor effects of TIIA on osteosarcoma both in vitro and in vivo. We also demonstrate that HGK-SAPK/JNK-Jun signal axis results in induction of TIIA-mediated autophagy and osteosarcoma growth inhibition, and their recruitment to SESN2 promoter leads to SESN2/AMPK-α activation. Understanding the reprogramming of cell death associated with cellular metabolic networks induced by TIIA in osteosarcoma may offer a novel antitumor drug candidate and aid in the development of novel approaches for future cancer therapy.

## Materials and methods

### Retrospective cohort study with Danshen

A retrospective study was conducted using the catastrophic illness database from years 1997 to 2011. Bone cancer patients who were diagnosed with ICD-9-CM code 170.x were identified from the catastrophic illness database covering 1997 to 2010, and were followed up until 31 December 2011. We recruited 159 patients with Danshen treatment and 673 non-Chinese herbal medicine (CHM) users all of whom were bone cancers patients. These patients were matched at baseline by sex, age (per 5 years), initial bone cancer year, and index year in a 1:1 ratio with a reference cohort and non-CHM user cohort. There were 22 patients for each cohort after frequency matching. Before frequency matching, a mean 41.54 ± 19.07 and 48.86 ± 24.57 years old for the used Danshen group and non-CHM user group, separately demonstrated (Supplementary Table 1). After frequency matching, a mean 51.68 ± 16.45 and 51.30 ± 16.17 years old for used Danshen group and non-CHM user group, respectively, resulted. Percentages of female and male were 54.55% and 45.45%, respectively. The 18–39 year, 40–59 year, and older than 60 year group proportions were 31.82%, 50.00% and 18.18%, respectively (Supplementary Tables S2). The primary outcome was overall survival rate during 14 years follow-up. All eligible patients were followed up from the index date to 31 December 2011, or death, or withdrawal from National Health Insurance, whichever occurred first.

### Cell culture

The human osteosarcoma cell line 143B and MG63 cells and human lung (carcinoma) epithelial cell line A549 cells were grown in Dulbecco’s modified Eagle medium (DMEM) supplemented with 10% fetal bovine serum (FBS) and antibiotics at 37 °C in a 5% CO_2_ incubator. To examine whether TIIA could induce autophagy, cells were treated with different doses of TIIA for 24 h.

### Constructs and transfection

For knockdown of SESN2, BECN1, and HGK, pGFP-V-RS plasmid encoding shRNA against human SESN2 (TG301755), BECN1 (TG314484), and HGK (TG320615) were purchased from OriGene (Rockville, MD). For luciferase reporter assays, the AP-1-Luc (60612) and SESN2-Luc (HPRM12429-PG02) were purchased from BPS Bioscience (San Diego, CA) and OriGene, respectively. The luciferase activities were measured using the Dual-Luciferase Reporter Assay System (Promega, Madison, WI, USA). The cells were stably transfected by using PolyJet^TM^ DNA In Vitro Transfection Reagent (SignaGen Laboratories), according to the manufacturer’s instruction.

### Collection of human tissue specimen

Normal bone tissue specimens were obtained from 13 patients with OA (at least 2 cm away from the joint) compared with tissue from 9 patients with osteosarcoma obtained from surgery. Informed consents were obtained from all individual cases, and the study was approved by the Institutional Research and Ethics Committee (CMUH104-REC3-110). The diagnosis of osteosarcoma was confirmed by pathologic results.

### Anchorage-independent growth assay

Anchorage-independent growth of osteosarcoma cells in the presence of TIIA was assayed by colony formation in soft agar. Cells were seeded 2 × 10^4^ cells/well in six-well plates containing 0.5% SeaPlaqueTM agarose (Lonza, Rockland, ME, USA) media. After 3 weeks, the numbers of colonies were counted at least 100 mm in the diameter.

### Detection of intracellular ROS

Intracellular ROS were evaluated by determining the level of hydrogen peroxide (H_2_O_2_) using a DCFDA (Sigma) fluorescent probe. In the presence of H_2_O_2_, DCFDA was converted into 2, 7-dichlorodifluoroscein, which could be detected by flow cytometry. After being treated with TIIA, the cells were incubated with 5 μM DCFDA for an additional 30 min, followed by washing and resuspending in phosphate-buffered saline (PBS). Detection of mitochondrial superoxide was achieved by using MitoSOX™ Red (Thermo Scientific, USA) superoxide indicator and measured by flow cytometry. 143B cells were seeded at a density of 6 × 10^5^ cells in six-well plates and allowed to attach for 24 h. The fluorescence was detected using a BD Biosciences FACScan system.

### Quantification of AVO development

The cells were stained with acridine orange, the cytoplasm and nucleolus fluoresce bright green and dim red, respectively, whereas acidic compartments fluoresce bright red. The prevalence of autophagic cells was also detected by evaluation of the development of AVOs, a marker of autophagy. Cells were stained with acridine orange for 17 min, removed from the plate with trypsin-EDTA, and collected in PBS containing 10% FBS. Green and red fluorescence emissions from 10^4^ cells illuminated with blue (488 nm) excitation light were measured with a BD Biosciences FACScan system (San Jose, CA) using CellQuest software. The red:green fluorescence ratio for individual cells was calculated and statistically analyzed.

### Intracellular ATP content

Cells were harvested in a reaction buffer containing 20 mM glycine, 50 mM MgSO4, and 4 mM EDTA (pH 7.4) and sonicated. ATP levels were measured by luciferase/luciferin-mediated assays. Briefly, 20 μl of the sample was mixed with 5 μl of luciferase-luciferin solution (Thermo Labsystems Oy, Helsinki, Finland) and the intensity of the emitted light was measured using a plate reader assay. The amount of ATP production was determined with the use of a standard curve constructed using 10–100 pmol ATP. Protein content was determined with bicinchoninic acid assay (Pierce, Rockford, IL, USA).

### Mitochondrial membrane potential determination with rhodamine 123

Mitochondrial membrane potential was measured using rhodamine 123 (Molecular Probes). Cells were cultured in DMEM without phenol red containing 3 μM rhodamine 123 for 15 min. The cells were then washed two times with DMEM without phenol red and resuspended in PBS. Depolarization of mitochondrial membrane potential caused rhodamine 123 to leak out of mitochondria into the cytosol where rhodamine 123 was unquenched, which increased fluorescence. The stained cells were analyzed by BD Biosciences FACScan system (San Jose, CA, USA).

### Animal

All experiments were done under Institutional Animal Care and Use Committee approval at China Medical University (Taichung, Taiwan) (2017–077). NOD/SCID (NOD CB17-Prkdcscid/NcrCrl, male, 5 weeks of age) mice were obtained from BioLASCO Taiwan Co., Ltd. All mice were housed under a setting of 12 h light/dark cycle at 22 ± 1 °C, 55% humidity, and fed with water and food provided at regular times. During the entire maintenance period, all mice were permitted free cage activity without joint immobilization. The initial body weights of the mice were between 20 and 23 g. After subcutaneous injection of 143B osteosarcoma cells into the back of NOD/SCID mice, the mice were treated with or without TIIA (20 mg/kg). TIIA was diluted in DMSO:ethanol:normal saline:hydroxypropyl-beta-cydodextrin = 1:3:3:3 and heated to 60 °C before injection mice. Seven days after 143B osteosarcoma cell injection, IP injection with TIIA was carried out every other day followed by sacrifice at day 45 of tumor cell inoculation. The tumors were removed, weighed, and fixed for use in immunohistochemical experiments. All experiments were carried out using five mice in each group in three independent experiments.

### Chromatin immunoprecipitation assay

Chromatin immunoprecipitation (ChIP) was performed using the Pierce Magnetic ChIP Kit from Thermo Scientific (Rockford, Illinois, USA) according to the manufacturer’s instructions. Jun-associated chromatin fragments were immunoprecipitated using anti-Jun antibody (Cell Signaling Technology). DNA was purified and subjected to PCR analysis. The fragment of the SESN2 promoter region was amplified with the primers listed in Supplementary Table S3 .

### Histological examination

All of the tumor mass dissected from mice were weighed and fixed in 4% paraformaldehyde (pH 7.5) for 4 h and then processed for paraffin embedding according to standard histological procedures. Sections with thickness of 4 μm were prepared and stained with H&E. For H&E staining, paraffin-embedded sample slides were de-paraffinized, hydrated, and then stained with hematoxylin for 1 min. After rinse, the slides were stained with eosin for 5 min, rinsed, and sealed with coverslips. The slides were counterstained with hematoxylin and mounted. To determine the effect of TIIA on expression of LC3B, P62, BECN1, Bcl-2, ATG5, ATG7, JNK1, class III PI3L, SESN2, LKB1, and p-AMPK-a by immunohistochemistry, the slides were blocked in 5% bovine serum for 15 min, followed by incubation with the primary antibody at 4 °C overnight in a moist chamber. The sections were then incubated with the corresponding secondary antibodies. The antigen-antibody complex was detected by Dako Liquid DAB + Substrate-Chromogen System (Dako, Carpinteria, CA). All slides were examined under light microscopy. We calculated IHC scores according to positive cells and intensity of staining products as described in previous reports^15^.

### RNA isolation and quantitative reverse transcription-polymerase chain reaction

Total RNA was extracted by a single-step phenol–chloroform–isoamyl alcohol extraction procedure modified from the protocol previously described^3^. Total RNA was isolated from cells by using the Trizol reagent protocol (Sigma). cDNA was synthesized using M-MLV Reverse Transcriptase (Promega, Madison, WI, USA). For real-time PCR analysis, reverse transcription was performed using 1 μg of total RNA and oligo (dT) primer in a 20 μl reaction according to the manufacturer’s protocol (Applied Biosystems, Foster City, California, USA). Real-time PCR was performed using the Mx3005 qPCR system (Stratagene, La Jolla, CA, USA) with SYBR green (Applied Biosystems, Foster City, CA, USA). The relative abundance of each mRNA was calculated using the ΔΔCt method and normalized to GAPDH. Primers are listed in Supplementary Table S3.

### Western blot analysis

The tumor masses and 143B cells cultured with or without TIIA were harvested and total cell protein was extracted using whole-cell lysis buffer. The protein concentrations were determined by the Bradford method (Bio-Rad, CA, USA). Samples with equal amount of protein were subjected to 8–15% sodium dodecyl sulfate polyacrylamide gel electrophoresis and transferred onto a polyvinylidenedifluoride (Millipore, Bedford, MA, USA) membrane. The membrane was incubated at room temperature in blocking solution (5% nonfat milk) for 1 h followed by incubation for 2 h in blocking solution containing an appropriate dilution of anti-LC3B, -P62, -SESN2, -HIF-1α, -castalase, -MnSOD, -GPX1, -Beclin 1, -ATG5, -ATG7, -JNK1, -p-SAPK/JNK, -c-Jun, -p-c-Jun, -PI3K 110λ, -p-PI3K p85/p55, -PI3K class III, -Akt, -p-Akt, -AMPK, -p-AMPK, -mTOR, and -p-mTOR antibody (Cell Signaling Technology, USA). After washing, blots were then probed with appropriate secondary horseradish peroxidase-conjugated secondary antibodies (Jackson ImmunoResearch, West Grove, PA) and detected by an ECL detection system (Millipore) and scanned by MultiGel-21 (Top Bio, Taiwan). β-actin served as internal control.

### Immunofluorescence microscopy

 For immuno-staining, cells were fixed in 4% paraformaldehyde for at least 10 min. Cells were then blocked with 3% bovine serum albumin (BSA) in PBS at 37 °C for 60 min and incubated with primary antibody (antibody diluted in 3% BSA) at 4 °C overnight. Cells were then washed three times with PBS and incubated with secondary antibody at 37 °C for 60 min in the dark. The cells were washed three times with PBS and stained with DAPI, air-dried at 4 °C in the dark, washed three times with PBS. After mounting in fluoromount media (Sigma-Aldrich Co. LLC, St. Louis, MO, USA), the slides were visualized under a confocal microscopy.

### TUNEL assay

Cell death in sections was assessed using In Situ Cell Death Detection Kit (ROCHE, Indianapolis, USA). Following fixation and permeabilization, sections were treated with a terminal deoxynucleotidyltransferase according to the manufacturer’s instructions.

### Statistical analysis

All statistical analyses were performed using GraphPad Prism statistical software (version 6, GraphPad Software, Inc., San Diego, CA). Results were represented as means ± standard deviation. One-way analysis of variance was carried out when multiple comparisons were evaluated. Values were considered to be significant at *P* < 0.05. All experiments were repeated independently at least three times.

## Electronic supplementary material

Supplementary Tables (S1-S3)

Supplementary Figure 1

Supplementary Figure 2

Supplementary figure legends
