# Supplementary material for: HGK-sestrin 2 signaling-mediated autophagy contributes to antitumor efficacy of Tanshinone IIA in human osteosarcoma cells
Source: Cell Death Dis. 2018 Sep 26;9(10):1003. doi: 10.1038/s41419-018-1016-9 (PMC6158215; doi:10.1038/s41419-018-1016-9)
Supplement: Supplementary file 1 — Supplementary Tables (S1-S3) [file 41419_2018_1016_MOESM1_ESM.pdf]

Supplementary Tables

Supplementary Table 1. Characteristics of bone cancer patients according to use of Chinese herb and non-used before frequency matching.

Variable	Bone cancer patients				*p*-value
	Non-TCM (n =673)		Using Danshen (n =159)		
	n	%	n	%	
**Gender**					<.0001
Female	234	34.77	87	54.72	
Male	439	65.23	72	45.28	
**Age group**					<.0001
0-18	124	18.42	22	13.84	
18-39	115	17.09	51	32.08	
40-59	217	32.24	18	11.32	
≥60	217	32.24	68	42.77	
Mean±SD (years)[a]	48.86(24.57)		41.54(19.07)		<.0001
**Follow time, years (mean, median)**	1.64(0.67)		4.13(3.07)		
**Interval between onset of cancer and the first using TCM date, day (median)**	459(175)		1326(898)		<.0001

*Chi-Square Test;[a] t test

Supplementary Table 2. Characteristics of bone cancer patients according to use of Chinese herb and non-used after frequency matching.

Variable	Bone cancer patients				*p*-value
	**Non-TCM (n =22)**		**Using Danshen (n =22)**		
	**n**	**%**	**n**	**%**	
**Gender**					0.99*
Female	12	54.55	12	54.55	
Male	10	45.45	10	45.45	
**Age group**					0.99*
18-39	7	31.82	7	31.82	
40-59	11	50.00	11	50.00	
≥60	4	18.18	4	18.18	
Mean±SD (years)[a]	51.30 (16.17)		51.68 (16.45)		0.9423[a]
**Follow time, years (mean, median)**	1.16 (0.62)		4.65 (2.46)		
**Interval between onset of cancer and the first using TCM date, day (median)**	157		194		0.8392[a]

*Chi-Square Test;[a] t test

Supplementary Table 3. Sequencing of primers.

Primers qPCR

**Human Gene**	**Forward**	**Reverse**
SESN2	5'- GAC CAT GGC TAC TCG CTG AT -3'	5'- GCT GCC TGG AAC TTC TCA TC -3'
HIF-1α	5'- AAA CTT GGC AAC CTT GGA TTG G -3'	5'- TCC GTC CCT CAA CCT CTC AG -3'
catalase	5'- ACT TCT GGA GCC TAC GTC CT -3'	5'- TCT GGA ATC CCC CGA TCA CT -3'
MnSOD	5'- AGC GGC TTC AGC AGA TCG -3'	5'- AGA TAC CCC AAA ACC GGA GC -3'
GPX1	5'- GCG GCC CAG TCG GTG -3'	5'- CAG AGG GAC GCC ACA TTC TC -3'

Primers ChIP

**Promoter**	**Forward**	**Reverse**
SESN2	5′- AAA AGG GTC AGA TAA AAC AT -3′	5′- TTA GTA AAT AGA GAC AGG GT -3′
