# Supplementary figures and images for: HGK-sestrin 2 signaling-mediated autophagy contributes to antitumor efficacy of Tanshinone IIA in human osteosarcoma cells
Source: Cell Death Dis. 2018 Sep 26;9(10):1003. doi: 10.1038/s41419-018-1016-9 (PMC6158215; doi:10.1038/s41419-018-1016-9)

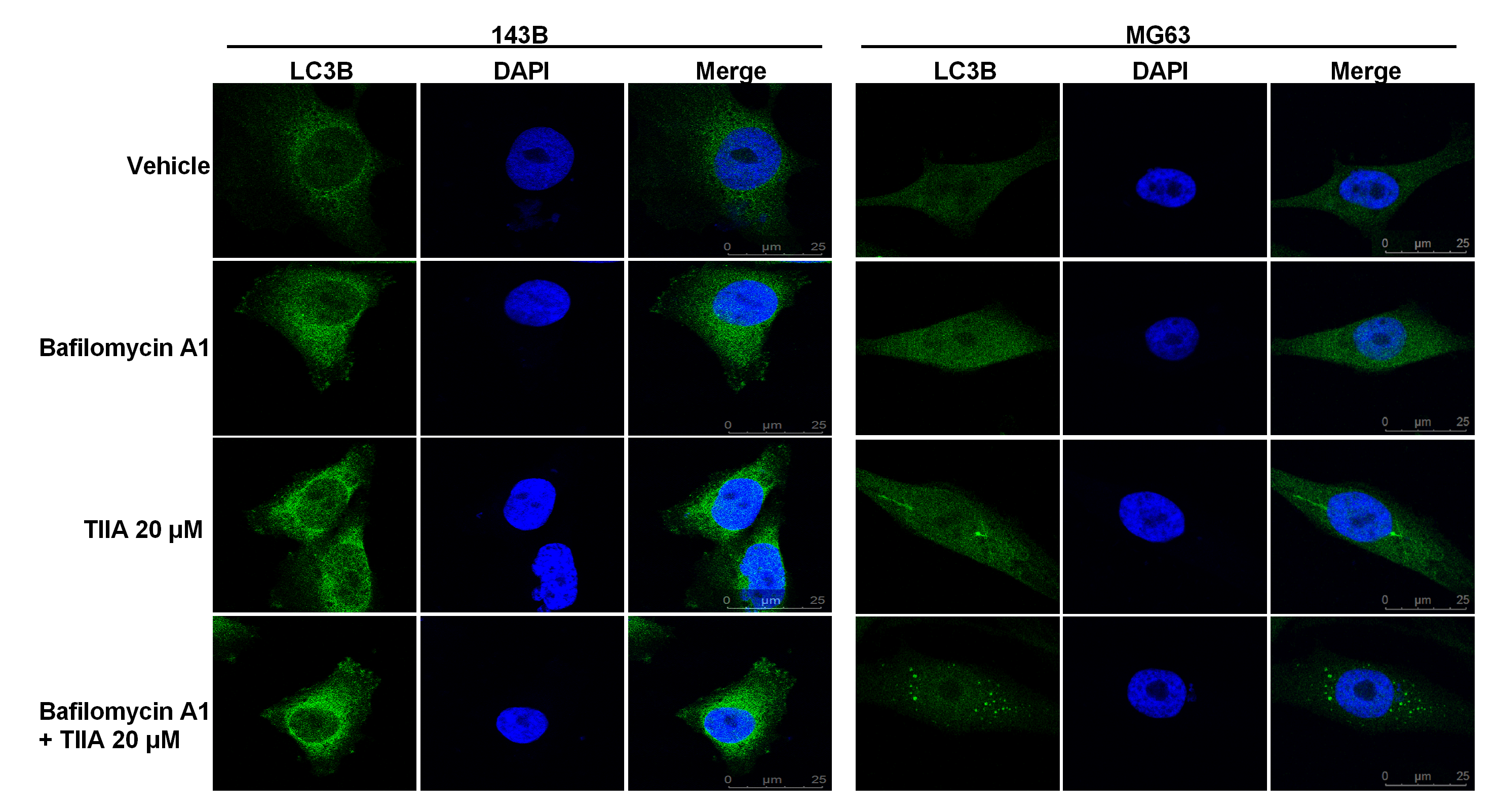

Supplement: Supplementary file 2 — Supplementary Figure 1 [file 41419_2018_1016_MOESM2_ESM.tif]

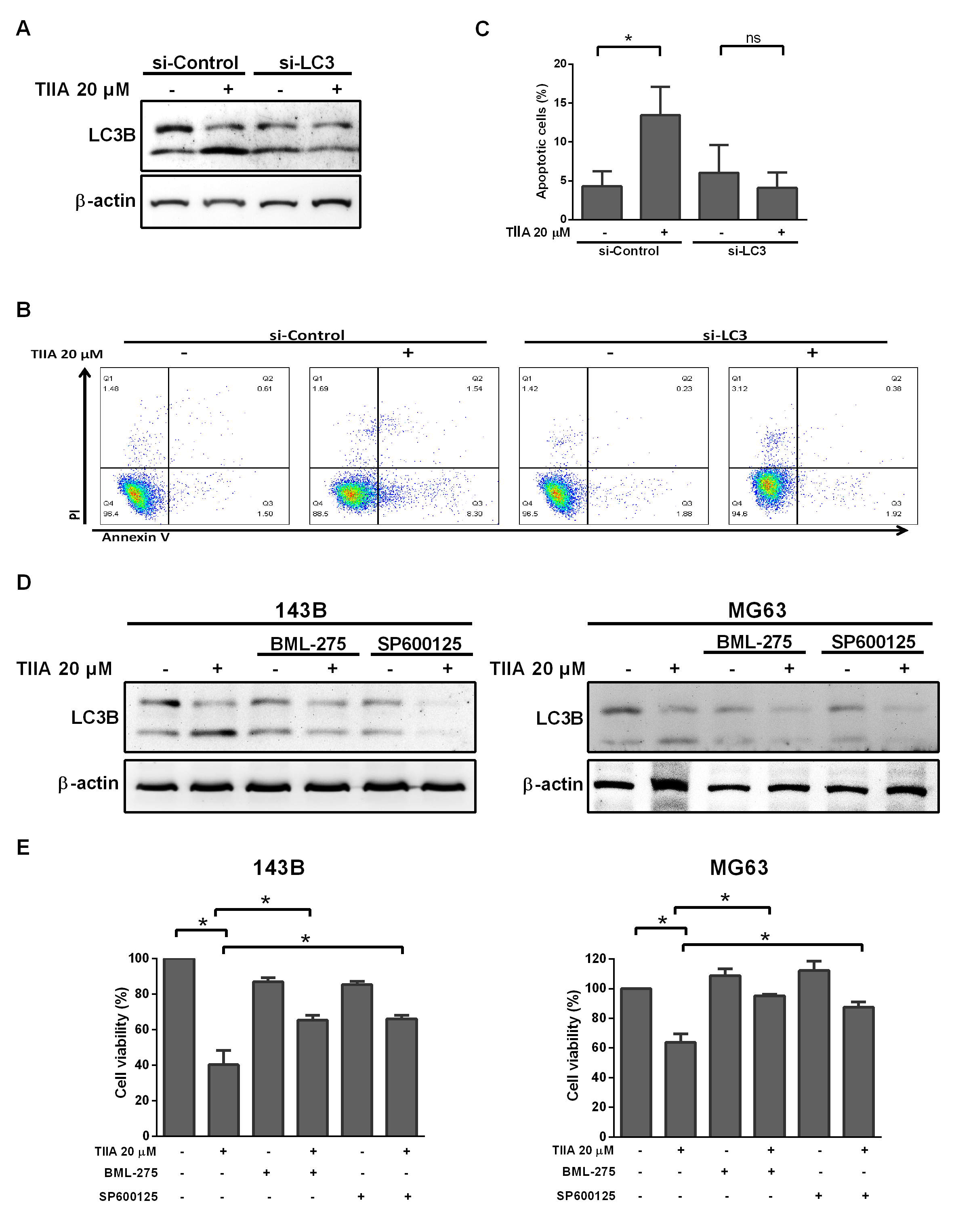

Supplement: Supplementary file 3 — Supplementary Figure 2 [file 41419_2018_1016_MOESM3_ESM.tif]
