# Supplementary material for: HGK-sestrin 2 signaling-mediated autophagy contributes to antitumor efficacy of Tanshinone IIA in human osteosarcoma cells
Source: Cell Death Dis. 2018 Sep 26;9(10):1003. doi: 10.1038/s41419-018-1016-9 (PMC6158215; doi:10.1038/s41419-018-1016-9)
Supplement: Supplementary file 4 — Supplementary figure legends [file 41419_2018_1016_MOESM4_ESM.docx]

**Supplementary materials**

**Table S1** Characteristics of bone cancer patients according to use of Chinese herb and non-used before frequency matching.

**Table S2** Characteristics of bone cancer patients according to use of Chinese herb and non-used after frequency matching.

**Table S3** Sequencing of primers.
